# Supplementary material for: Stacking Model‐Based Classifiers for Dealing With Multiple Sets of Noisy Labels
Source: Biom J. 2025 Mar 12;67(2):e70042. doi: 10.1002/bimj.70042 (PMC11898607; doi:10.1002/bimj.70042)
Supplement: Supplementary file 1 — Supporting Information [file BIMJ-67-e70042-s001.zip › density-based-ensemble-model-main/competitors_real_data/netlab-master/netlab-master/help/glmfwd.htm]

Netlab Reference Manual glmfwd


# glmfwd

## Purpose

Forward propagation through generalized linear model.

## Synopsis

```
y = glmfwd(net, x)
[y, a] = glmfwd(net, x)
```

## Description

`y = glmfwd(net, x)` takes a generalized linear model
data structure `net` together with
a matrix `x` of input vectors, and forward propagates the inputs
through the network to generate a matrix `y` of output
vectors. Each row of `x` corresponds to one input vector and each
row of `y` corresponds to one output vector.

`[y, a] = glmfwd(net, x)` also returns a matrix `a`
giving the summed inputs to each output unit, where each row
corresponds to one pattern.

## See Also

`glm`, `glmpak`, `glmunpak`, `glmerr`, `glmgrad`

---

**Pages:**
Index


---

Copyright (c) Ian T Nabney (1996-9)
